# Alternative Splicing of the NF-Y Subunit, NF-YA, in Neuroblastoma Phenotype Heterogeneity

**DOI:** 10.3390/cancers18111839

**Published:** 2026-06-04

**Authors:** Ilaria Martelli, Lucia Anna-Maria Cappabianca, Maddalena Sbaffone, Antonietta Rosella Farina, Andrew Reay Mackay

**Affiliations:** Department of Applied Clinical Sciences and Biotechnology, University of L’Aquila, 67100 L’Aquila, Italy; ilaria.martelli@graduate.univaq.it (I.M.); cappabianca@univaq.it (L.A.-M.C.); maddalena.sbaffone@univaq.it (M.S.); antonietta.farina@univaq.it (A.R.F.)

**Keywords:** neuroblastoma, phenotype heterogeneity and plasticity, NF-Y, NF-YA alternative splicing, drug resistance

## Abstract

Neuroblastomas (NBs) are aggressive, therapy-resistant embryonal tumors of neural crest origin, which despite low mutational burdens exhibit high intra-tumoral heterogeneity characterized by adrenergic, noradrenergic, mesenchymal and cancer stem cell (CSC)-like subpopulations. These phenotypes exhibit interconverting plasticity that reflect both stage of transformation during sympathoadrenal development and conditions within the tumor microenvironment. Chemotherapeutic agents promote adrenergic-to-mesenchymal conversion in NBs, which underpins drug resistance, post-therapeutic relapse, metastatic progression, and the plateauing of responses to advances in multimodal therapy. Improved understanding of the molecular mechanisms that regulate NB phenotypic plasticity is essential for identifying novel prognostic markers and potential therapeutic targets. In this article, following introductions into NB, molecular regulation of NB phenotypic plasticity, and the NF-Y transcription factor and its role in development and differentiation, we focus on alternative NF-YAl, NF-YAs and NF-YAx splicing of the NF-Y subunit, NF-YA, and the potential influence that different NF-YA isoforms have on NF-Y function and the NF-Y-transcription factor networks that impact NB cell phenotypes. Particular attention is paid to the novel extra short-form NF-YAx isoform, originally detected as the exclusive NF-YA isoform in a non-*MYCN* amplified advanced stage 3 NB. This isoform is also induced by doxorubicin in non-Myc amplified SH-SY5Y NB cells and is involved in doxorubicin cytotoxicity. Despite high cytotoxicity, however, NF-YAx selects a resistant subpopulation with mesenchymal/neural crest stem cell-like identity, unveiling a doxorubicin-induced NF-YAx-dependent resistance mechanism, with potential to influence post-therapeutic relapse and disease progression. Therefore, evaluating alternative NF-YA splicing, and especially NF-YAx expression, in advanced stage and post-therapeutic relapsed NBs, may be of both prognostic and therapeutic significance.

## Simple Summary

Alternative splicing of the NF-Y transcription factor component, NF-YA, and expression of the novel NF-YAx isoform, in particular, are potential key regulators of cellular phenotypic plasticity, stemness, stress and drug resistance in neuroblastoma. NF-YAl, NF-YAs and NF-YAx isoforms cause functional NF-Y modifications that alter the mRNA expression of core phenotype regulators that may result in less-aggressive, more-adrenergic (NF-YAl) or resistant more-aggressive mesenchymal/neural crest stem cell-like phenotypes (NF-YAs and NF-YAx), with potential to impact response to therapy, post-therapeutic relapse and progression. Differential expression of these three NF-YA isoforms in neuroblastomas may, therefore, be of prognostic and therapeutic significance.

## 1. Introduction

Neuroblastomas (NBs) are aggressive, therapy-resistant embryonal, immunologically “cold” tumors that arise from cells of neural crest origin, directed along the sympathoadrenal lineage. They represent the most common extracranial solid tumor of childhood and account for ≈15% of pediatric cancer-related deaths. Although NBs may arise at different sites along the developing sympathetic nervous system, the majority arise within the adrenal medulla (≈47%), and to a lesser degree within the abdomen (≈24%), thorax (≈15%), pelvis and neck (≈3%). Histological NB subtypes include undifferentiated tumors that exhibit <5% differentiated cells and differentiating tumors exhibiting >5% differentiated cells. Favorable or unfavorable prognoses are determined by age, degree of differentiation and mitosis-karyorrhexis index. Localized tumors are classified as L1 or L2 based upon the absence or presence of image-defined risk factors, respectively; metastatic tumors in infants less than 18 months old are classified as MS, and the rest are classified as M. These classifications are used to allocate patients to high-risk, intermediate-risk, low-risk and very-low-risk groups based upon prognostic molecular and histological characteristics [1,2,3,4,5,6].

Tumor heterogeneity is a complex phenomenon driven by cell-intrinsic factors including genomic instability, somatic mutations, epigenetic changes and plastic gene expression, and by extrinsic factors within the tumor microenvironment (TME). Extrinsic factors include infiltrating host immune, inflammatory and stromal cells, an ever changing tumor microvasculature, and stresses caused by fluctuating nutrient availability, chronic and cyclic episodes of hypoxia, reoxygenation with re-nutrition, and therapeutic agents. These different components co-exist, interact, are constantly modified, and activate adaptive stress responses in tumor and tumor infiltrating host cells, resulting in the selection of genotypes and phenotypes better equipped to survive within each specific TME region. Within therapeutic settings, phenotypic conversion induced or selected by chemotherapeutic agents facilitates acquisition of drug resistance, which underpins post-therapeutic relapse and subsequent disease progression [7,8,9,10].

## 2. Phenotypic Heterogeneity and Plasticity in NB

NBs, despite low mutational burdens, exhibit remarkably high inter- and intra-tumoral heterogeneity that impact therapeutic resistance, immunogenicity, post-therapeutic relapse, metastatic disease progression and outcome [11,12,13,14,15,16,17,18,19,20,21,22,23,24,25,26]. This heterogeneity was originally described as neuronal (N), intermediate (I), substrate adherent (S) and stem cell-like phenotypes in NB cell lines [12,27,28]. Advances in gene expression and epigenetic profiling have improved understanding of NB phenotypic heterogeneity and plasticity. Two predominant NB cell identities are now considered to regulate NB behavior, characterized as partially differentiated adrenergic and undifferentiated mesenchymal phenotypes that exhibit interconvertible plasticity, and are detected in human NBs. These phenotypes are determined by specific Core transcriptional circuitries, exhibit mutation-independent interconversion through epigenetic regulation and transcriptional reorganization, provide selective advantages within specific TMEs, and regulate aggressivity and responses to therapy, post-therapeutic relapse, metastatic progression and outcome [7,8,9,10,11,12,13,14,15,16,17,18,19,20,21,22,23,24,25,26,27,28,29,30,31,32,33,34]. Chemotherapeutic agents promote adrenergic-to-mesenchymal phenotype conversion, which is considered a critical determinant in the acquisition of therapeutic resistance and subsequent post-therapeutic relapse, through the selection and/or generation of a rapidly expanding, drug-resistant mesenchymal population. This underpins the plateauing of responses to advances in multimodal therapy in high-risk disease. Different NB phenotypes may also represent distinct cell types selected by differences in responses to the TME and therapy in terms of elimination, survival and proliferation [22,23,29,35].

**Figure 1:**
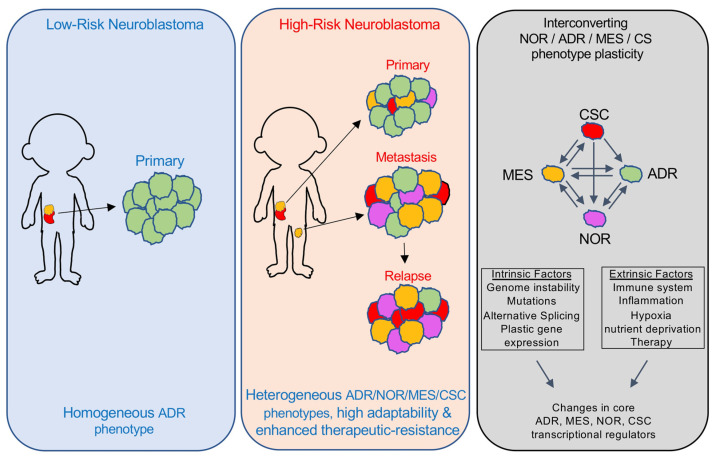
Heterogeneity in NBs is influenced by intrinsic and extrinsic factors and impacts, response to therapy, acquisition of therapeutic resistance, post therapy relapse and metastatic progression. Low-risk NBs are relatively homogeneous (**left panel**), whereas advanced stage metastatic and relapse NBs (**middle panel**) contain adrenergic (ADR), noradrenergic (NOR), mesenchymal (MES) and cancer stem cell (CSC) components that exhibit interconverting plasticity enhancing survival adaptability within the stressful tumor microenvironment, involved in therapeutic resistance, relapse and progression (**right panel**).

In general, adrenergic NB cells are more differentiated along the sympathetic neuronal lineage, less aggressive and more sensitive to therapy, whereas mesenchymal NB cells, which exhibit overlapping neural crest stem cell (NCSC)-like features, are more therapy-resistant, invasive and malignant [1,22,29,33,35]. Low-risk stage 1 and 2 primary NBs exhibit predominantly more differentiated adrenergic phenotypes, resemble more differentiated neuronal cells, and despite heterogeneity maintain this phenotype in association with PHOX2B, GATA3 and ASCL1 expression. Advanced stage 3 and 4 NBs also exhibit predominantly adrenergic phenotypes but also contain variable sub-populations of undifferentiated, migratory, mesenchymal NSCS-like cells. This subpopulation becomes enriched in post-therapy relapsed tumors and plays a critical role in the progression of stage 3 and stage 4 NBs due to enhanced resistance to chemo- and radio-therapy, favoring therapeutic promotion of more CSC-like, stress-resistant, aggressive and metastatic behavior [23,33,34] (Figure 1).

NB adrenergic and mesenchymal phenotypes are driven by distinct core regulatory transcriptional factor networks, epigenomic and transcriptomic states. Adrenergic core transcriptional circuitry includes PHOX2A/PHOX2B, HAND1/HAND2, TBX2, ASCL1, ISL1, FOXM1 and GATA3 transcription factors, several of which also regulate sympathetic nervous system development [13,14,18,29,30,31,32,33,34]. Core adrenergic regulators also interact with the MYCN oncoprotein [13,14], which is amplified in between 20 and 50% of high risk NBs [36]. This is exemplified by the *TBX2* gene on chromosome 17q, frequently gained in high-risk NBs, that interacts with MYCN to increase FOXM1 expression driving proliferation and survival [37,38]. The core adrenergic transcription factor ASCL1 is a selective NB dependency gene [39], is expressed by sympathetic progenitors, regulates HAND2, ISL1, PHOX2B, GATA3 and TBX2 expression [13,14], and is involved in neuronal differentiation [40]. The adrenergic phenotype also depends upon levels of MYCN expression, as physiological MYCN expression promotes predominant transcription of genes with strong E-boxes, whereas a high level of MYCN expression also activates genes with weak E-boxes, resulting in tumor-specific gene expression in *MYCN* amplified NBs [41,42]. In a zebra fish NB model, the MCYN transcriptional co-regulator LIM-domain only 1 (LMO1) accelerates NB initiation and progression, and interacts with core adrenergic transcription factors to establish adrenergic identity [43]. In human NB cells, DNA topoisomerase 2-beta (TOP2B) promotes and maintains the adrenergic phenotype and suppresses the mesenchymal phenotype [44].

The mesenchymal NB phenotype is less well defined but associated with the expression of PRRX1, AP-1, IRF1/IRF2/IRF3, RUNX1/RUNX2, MEOX1/MEOX2, SIX1/SIX4, SOX9, SMAD3, WWTR1 and NOTCH3 transcription factors. Adrenergic-to-mesenchymal phenotype conversion can be induced by reducing core adrenergic transcription factor expression [13,14,15,16,25,26,29,45,46], and is promoted by forced PRRX1 expression, by enhancing SNAI2 and repressing PHOX2B and DBH expression [12,24,25,26,30,46]. PRRX1-positive mesenchymal NB cells are enriched in post-therapeutic relapsed NBs, consistent with drug-induced adrenergic-to-mesenchymal conversion or mesenchymal enrichment, with phenotypic plasticity confirmed by reversion following treatment [23,47]. This is corroborated by the phenotypic interconversions exhibited by NB cell lines in vitro and in vivo. PRRX1 silencing, however, does not result in mesenchymal-to-adrenergic reconversion, implicating additional factors in phenotype reversal plasticity [12]. Overexpression of NOTCH3 also promotes NB cell adrenergic-to-mesenchymal conversion by repressing core adrenergic regulatory factors, and is considered to be a master regulator of this process [33,45]. GATA3 gene knock-out promotes NB cell adrenergic-to-mesenchymal conversion in association with reduced PHOX2A, PHOX2B and HAND2 expression, enhances drug resistance, promotes invasive behavior, and is considered to be a critical component of phenotypic plasticity [46]. In non-*MYCN*-amplified SH-SY5Y NB cells, silencing TOP2B expression promotes adrenergic-to-mesenchymal conversion by down modulation of ≈47% of adrenergic phenotype-associated gene expression and upregulation of ≈38% of mesenchymal phenotype-associated gene expression, consistent with a critical role in maintaining the adrenergic phenotype and suppressing the mesenchymal phenotype [44]. In addition to this, CX-5461 TOP2B inhibitor induces DNA damage and apoptosis in NB cells [48], suggesting that TOP2B inhibition selects mesenchymal NB cells by eliminating adrenergic counterparts. The CD44 hyaluronic acid receptor is considered to be a specific marker of the mesenchymal NSCS NB phenotype, and NB cell CD44 expression is influenced by both culture and TME conditions [23,47]. In a xenograft NB model, CD44-positive mesenchymal NB cells are driven back into the adrenergic phenotype by the TME, and CD44-positive cells isolated from xenografts, exhibiting a predominant adrenergic phenotype, reconvert back to the mesenchymal phenotype upon culture in vitro. This indicates that adrenergic identity does not eliminate reversible plasticity [47]. NB phenotypic plasticity is also regulated at the epigenetic level in association with altered expression of the SW1/SNF chromatin remodeling complex component ARID1A, which associates with decreased survival and is repressed in ≈11% of NBs. ARID1A depletion in *MYCN*-amplified NB cells promotes adrenergic-to-mesenchymal conversion, and enhances drug resistance and invasive capacity, in association with altered chromatin H3K27ac marks at PHOX2B and fibronectin FN1 loci. This results in the repression of PHOX2B expression and enhanced expression of the mesenchymal marker fibronectin [26,49]. In general, mesenchymal NB phenotypes are more resistant to chemotherapeutic agents, such as cisplatin, doxorubicin and etoposide, and are considered to be more invasive and more metastatic than adrenergic phenotypes. Furthermore, adrenergic-to-mesenchymal NB cell conversion is promoted by chemotherapeutic agents [22,23,29]. This, however, is also debatable, as the adrenergic SH-SY5Y cell line [28,50] is significantly more aggressive than the mesenchymal SH-EP cell line, both of which are derived from the SK-N-SH cell line [28,50,51,52]. This is consistent with a recently described aggressive noradrenergic transitional intermediate NB phenotype, with sympathoadrenal development-associated gene expression, that exhibits rapid proliferation, enhanced dissemination and worse prognosis compared to mesenchymal counterparts [12,24,26].

It is clear that in NBs, the transition from adrenergic-to-mesenchymal NCSC-like phenotypes, or mesenchymal sub-population selection, is regulated by conditions within the TME that alter the expression of core phenotype transcriptional regulators, and plays a determining role in the acquisition of drug resistance, post-therapeutic relapse and metastatic progression. This makes the identification of patterns of regulator expression and markers of the different NB phenotypes important in prognosis and therapeutic decision making. In this regard, chemotherapeutic agents induce adrenergic-to-mesenchymal transition and select more resistant mesenchymal phenotypes that exhibit enhanced expression of inflammatory cytokines and antigen presenting proteins. This unveiling a potentially exploitable immunological Achilles heel through which chemotherapy could be combined with immunotherapy to eliminate both drug-sensitive adrenergic non-immunogenic and more-immunogenic mesenchymal components [29].

In this review, based upon this background, we examine the hypothesis that alternative splicing of the NF-YA component of the ubiquitous, heterotrimeric pioneer transcription factor NF-Y is also involved in regulating NB phenotypic heterogeneity and may influence drug-induced acquisition of therapeutic resistance, through the selection of mesenchymal/NCSC-like NB subpopulations.

## 3. NF-Y Transcription Factor

NF-Y is a ubiquitous heterotrimeric transcription factor, composed of NF-YA, NF-YB and NF-YC sub-units, that binds CCAAT-boxes upstream of gene transcriptional start sites in approximately 60–70% of human gene promoters and maintains the upstream borders of core promoters free of nucleosomes. It is the most abundant and arguably the primary, if not the only, CCAAT-box binding transcription factor in eukaryotes and is the most important determinant of transcription from promoters that exhibit precise locations of CCAAT-bos sequences in regulatory regions [53,54,55,56,57,58]. All three NF-Y subunits are required for CCAAT-box binding, NF-YB and NF-YC contain histone fold domains and form tight heterodimers that bind DNA in a nonspecific manner, but acquire high CCAAT-box binding affinity and specificity by associating with NF-YA. Both NF-YA and NF-YC contain large glutamine (Q)-rich sequences with transcriptional activating potential [59]. Upon interaction with DNA, the NF-YA helix A2 contacts the CCAAT- box and inserts into the DNA minor groove, which widens at the first adenine leaving the adjacent major groove available for binding other transcription factors. This permits NF-Y to synergize with a variety of general transcription factors that bind elements at conserved distances from the CCAAT-box, at 60 to 100 bases upstream of transcriptional start sites [60,61]. The importance of this spacing is illustrated by NF-Y regulation of the cellular response to ER stress. ER stress response genes are characterized by a single CCAAT-box separated from a CCACG box by nine nucleotides. Alteration of this spacing abolishes interaction-dependent transcriptional activity of NF-Y with the ER stress-response transcription factor ATF6, preventing ATF6-regulated ER stress response gene expression [61,62]. NF-Y regulation of gene expression is also enhanced by associations and interactions with general transcription factors, including Sp1 that binds GC-boxes in >80% of human gene promoters; cell-cycle-regulating E2Fs; CCAAT/enhancer-binding-protein C/EBP; SMAD 2/3 involved in TGFβ signaling, and E-box-binding MYC, ZHX1 and USAF1/2 transcription factors [63,64,65,66,67,68]. NF-Y also regulates alternative splicing through RBM25 and HNRNPLL RNA-binding proteins, and transcriptional repression through NuRD and DREAM repressive transcriptional complex components [69], and acts as a pioneer transcription factor by promoting chromatin accessibility during development [70].

**Figure 2:**
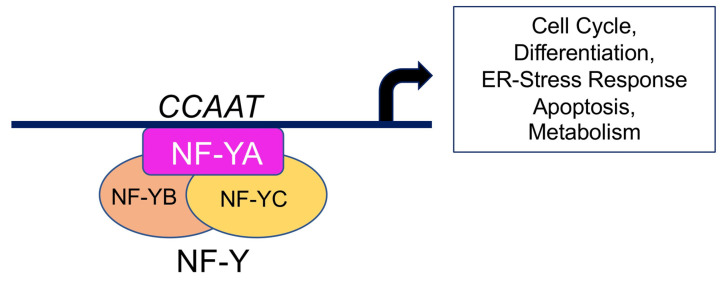
Diagram of the pioneer CCAAT-box binding heterotrimeric transcription factor NF-Y, composed of NF-YA, NF-YB and NF-YC subunits, that regulates the expression of genes involved in cellular functions, including proliferation, differentiation, ER stress, apoptosis and metabolism.

Genes regulated by NF-Y that contain functional CCAAT boxes are involved in cellular processes, including cell cycle S (e.g., PCNA, MYC and MCM4), G2 (e.g., CCNA1/2) and G2/M (e.g., CDC2, CCNB1, CCNB2, PLK and AURKB) phases; the cellular response to hypoxia (e.g., HIF1a and BIMI-1); the ER stress response and apoptosis (e.g., CHOP, ATF6, XBP1, TP53 and TP63); lipid metabolism (e.g., SREBP1, HMGCS1, HMGCR, SQLE, DHCR7, ACAC, FASN, SCD); glycolysis (e.g., PFKFB2/3/4, PGK 1/2, GAPDH, PKM, LDHA A/B/C, HK2 and ALDOB); and serine/glycine (e.g., PHGDH, PSAT1, PSPH, MTHFD2 and DHFR), glutamine (e.g., GLS) and polyamine/purine pathways (e.g., AMD1, ODC1, MTAP, AK2 and RRM1) [65,66,67,68,69,70,71] (Figure 2).

### 3.1. NF-YA and Its Alternative Splice Variant Isoforms

**Figure 3:**
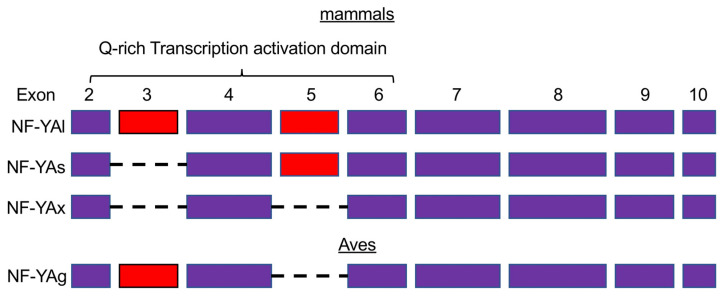
Representation of NF-YA splice variants in mammals and birds.

The NF-YA component of NF-Y is expressed as two major alternatively spliced isoforms in mammals, fully spliced long-form NF-YAl and exon 3-skipped short-form NF-YAs bearing a 28 amino acid deletion within the Q-rich transcriptional activation domain [72]. NF-YAl exhibits predominant expression in liver, brain and lung tissues, and in differentiated neurons, myocytes and fibroblasts. In contrast, NF-YAs exhibits predominant expression in the thymus, spleen and bone marrow, in murine and human embryonic stem cells and during early-stage murine embryo development. NF-YAs converts to predominant NF-YAl expression in stem-cell-derived embryoid bodies, upon differentiation, and during late-stage murine embryo development [35,73,74]. NF-YAs is also required for hematogenous stem cell maintenance and is reduced upon hematopoietic differentiation [75]. Both NF-YAl and NF-YAs are expressed by embryonic and fetal myoblasts, whereas differentiated muscle cells are characterized by exclusive low-level NF-YAl expression [73,74,76,77]. NF-YAs expression predominates in neural progenitors and switches to NF-YAl expression during neuronal differentiation, and NF-YAl is the predominant isoform expressed by murine striatal, cortex and hippocampus tissues [73,78,79,80]. Recently, an extra short-form NF-YAx (GenBank: OP894117.1) alternatively spliced isoform, exhibiting exons 3, 5 and partial 7-skipping, has been reported in primary human NBs, and during E12.5 through E14.5 stages of murine embryo developmental [35]. A fourth alternative splice variant, NF-YA*g*, exhibiting exon 5-skipping, has also recently been identified in Aves [81] (Figure 3).

Although not dealt with in this review, *NF-YC* also generates 50 kDa, 48 kDa and 37 kDa isoforms through differential promoter use and alternative splicing, that exhibit differences in Q-rich transactivating domains that influence NF-Y function within the context of alternative NF-YA splicing. The 50 kDa NF-YC isoform predominates in cells exhibiting predominant NF-YAs expression, whereas the 48 kDa isoform predominates in cells exhibiting predominant NF-YAl expression. This has implications for progenitor cell maintenance and differentiation, and the 37 kDa isoform plays a specific role in reducing the DNA damage response during proliferation [82].

### 3.2. NF-Y in Development and Differentiation

NF-Y acts as a pioneer transcription factor during murine development. In embryonic stem cells, NF-Y interacts with and regulates Oct4, Sox2, Klf4 and Nanog components of the stem cell core transcriptional program, and *NF-YA* knock-out is embryonically lethal in mice [73,74]. NF-Y initiates zygote-independent gene expression at the two-cell stage, prior to the appearance of h3K4me3 marks at the eight-cell stage, and regulates the zygotic gene-activation transposable element LTR12. This, combined with oocyte NF-YA, identifies NF-Y as a pioneer transcription factor and major driver of embryonic development [70,73,74,83].

In the hemopoietic system, NF-YAs overexpression in primitive stem cells reintroduced into bone marrow-ablated mice enhances mature myeloid precursor repopulation but halves differentiated cell numbers, in association with increased HoxB4, Lef-1, Notch-1 and Hes-1 expression. NF-YA expression is high in B and T lymphocytes, and *NF-YA* knockout does not influence the behavior of quiescent hematopoietic stem cells but causes apoptosis upon induction of proliferation. This unveils a role for NF-Y in exiting the quiescent stem cell state. Dominant negative _DN_NF-YA, which abrogates NF-Y DNA binding, reduces but does not impede early myeloid precursor differentiation, implicating NF-Y in the proliferation but not early differentiation of the hematopoietic lineage. Circulating monocytes express all NF-Y subunits whereas differentiated macrophages express only NF-YA [73,75]. In muscle differentiation and regeneration, all NF-Y subunits are expressed by proliferating skeletal muscle cells. Multinucleated differentiated muscle cells express little, if any, NF-YAl, implicating NF-Y in maintaining myocytes in a proliferating, non-differentiated state required for muscle regeneration. NF-YA expression is also essential for immature cardiomyocyte proliferation and cardiomyocyte physiology [73,76,77]. NF-Y is also involved in liver, pancreatic and adipocyte function. *NF-YA* knockdown in mice accelerates liver damage by de-regulating the ER stress response and reduces gluconeogenesis. In pancreatic b-cells, *NF-Y* knockout promotes a condition similar to type-2 diabetes, and in adipocytes, it de-regulates leptin expression resulting in leptin-reversible metabolic syndrome [84,85,86,87]. In nervous system development and function, NF-YAs is required for neuronal progenitor cell maintenance and cell cycle gene expression, whereas NF-YAl promotes neuronal differentiation and survival [73,78,79,80]. In mice, *NF-YA* deletion promotes neurodegeneration by de-regulating the ER stress-response, leading to misfolded protein accumulation within the ER and accelerated context-dependent neuronal death [88]. This is exemplified in Huntington’s disease, in which mutated Huntingtin poly-glutamines sequester NF-YA, reducing transcriptional activity and Hsp70 expression, which increases the accumulation of toxic poly-glutamine aggregates within the ER, leading to neurodegeneration [89]. NF-YA also regulates neurotransmitter, neuropeptide and G-protein coupled receptor activity in different neuronal subtypes, and loss of NF-YA expression compromises neuron synapse biology [78]. NF-YA also drives Sox expression in neuronal progenitors required for early stem cell differentiation events and differentiation into various neuronal subtypes, and is critical for both the maintenance and exit from neural and NSCS cell states [80].

### 3.3. Alternative NF-YA Splicing and the NB Phenotype

NF-YA expression is fundamental for zygote gene activation [73,90], and differences in the ratio of alternative NF-YAl and NF-YAs splice variants underpin the maintenance and exit from proliferating embryonic, neural and SCSC states [80]. Therefore, it follows that alternative NF-YA splicing may also be involved in regulating the interconverting plastic adrenergic/mesenchymal phenotypes that characterize NB heterogeneity, therapeutic response, relapse and progression [1,2,3,4,5,6]. In this regard, embryonic neural stem/progenitor cells exhibit predominant NF-YAs expression, whereas differentiated neurons exhibit predominant NF-YAl expression, and neuronal differentiation from progenitors is characterized by a switch from predominant NF-YAs to predominant NF-YAl expression [73,78,79,80].

In neural stem/progenitor cells, predominant NF-YAs expression, driven by a cell cycle-associated splicing program that promotes *NF-YA* exon 3 exclusion, must change to a differentiation-associated splicing program that promotes *NF-YA* exon 3 inclusion to increase NF-YAl expression and promote differentiation. Forced NF-YAl expression in mice reduces the number of differentiated neurons by suppressing neural progenitor proliferation, confirming the critical role of NF-YAs in neural stem/progenitor maintenance and proliferation that ensures sufficient progenitor numbers for nervous system development [80]. Transitions from alternative NF-YAs to NF-YAl splicing are likely to involve changes in RBM47, PTB-1, HuR and RBFOX2 splicing factor expression, which regulate cancer-associated epithelial-to-mesenchymal transition (EMT) [91], exemplified by NF-YAl promotion of EMT in breast cancers that exhibit low level claudin-1 expression [92].

In primary NBs, low-risk stage 1 and 2 tumors predominantly comprise cells with more differentiated adrenergic sympathoadrenal lineage-committed phenotypes. High-risk stage 3 and 4 tumors are also colonized by adrenergic phenotypes but contain smaller mesenchymal-like sub-populations that are considered to be the potential origins of therapeutic-resistance, post-therapeutic relapse and metastatic progression [11,16,20,25,34]. In a 30 patient cohort of primary human NBs, NF-YA mRNA was detected in 30% of stage 1 and 2 NBs, and in 40% of stage 3 and 4 NBs. NF-YAl and NF-YAs mRNA ratios were approximately equal in stage 1 NBs, in the majority of stage 2 NBs and in a minority of stage 3 and stage 4 NBs. Predominant NF-YAl mRNA expression was detected in the majority of NF-YA positive stage 4 NBs, whereas predominant NF-YAs mRNA expression was absent in stage 1 NBs, and was detected in individual stage 2, 3 and 4 NBs. The novel NF-YAx alternative splice variant was identified in this NB cohort, as the relatively highly expressed exclusive splice variant in a stage 3 non-*MYCN* amplified NB, and a minor splice variant in 2 of 10 (20%) stage 2 non-*MYCN* amplified NBs. NF-YAx cDNA was cloned from the stage 3 non-*MYCN* amplified NB, and was characterized as an in-frame novel exons 3, 5 and partial 7-skipped splice variant [35].

In NB cell lines, adrenergic non-*MYCN* amplified SH-SY5Y and *MYCN*-amplified SMS-KCNR, IMR32 and SK-N-BE CHP-126, LAN-1, LAN-5 NB cell lines, used to model highly aggressive high-risk NB [12,26,28,50,93,94,95], exhibited either predominant NF-YAs or similar levels of NF-YAl and NF-YAs mRNA expression in vitro [35]. In contrast, less-aggressive mesenchymal SH-EP NB cells lines, derived from the same parental SK-N-SH cell line as SH-SY5Y cells, exhibited high-level exclusive NF-YAl mRNA and protein expression. This supports association between equal or predominant NF-YAs expression with more aggressive adrenergic phenotypes and NF-YAl with less-aggressive mesenchymal phenotypes [35,50,51,52], linking increased NF-YAs expression to more-differentiated states and NF-YAl with less-differentiated states. This corroborates a report associating NF-YAl expression with EMT in claudin low breast cancers [92], and reports that an intermediate noradrenergic phenotype may represent the most aggressive NB phenotype [12,24,26]. However, this contrasts with reports that neuronal differentiation neural progenitors and NB cells associates with switch from NF-YAs to NF-YAl expression [73,79].

None of these NB cell lines, however, constitutively express the NF-YAx splice variant [35], excluding NF-YAx in the regulation of NB phenotypes under normal, non-stressed conditions. However, in non-*MYCN* amplified SH-SY5Y cells, NF-YAx expression is induced by the DNA-damaging chemotherapeutic agent doxorubicin. This cell line is one of the most widely used in vitro models of non-*MYCN* amplified NB, and is typically used as a human cellular model to study the biology and responses of high risk non-*MYCN* amplified NB to multi-model induction chemotherapy [96]. For this reason, the SH-SY5Y cell line was chosen to model the potential effects of NF-YAx detected in non-*MYCN* amplified stage 2 and stage 3 NBs.

Doxorubicin-induction of NF-YAx expression in SH-SY5Y cells was associated with late-stage cell death but was not induced by agents that promote either ER stress or hypoxia. This implicates the DNA-damage response in promoting a splicing program that facilitates NF-YAx expression, and implicates NF-YAx in doxorubicin-induced cytotoxicity [35]. Indeed, transient NF-YAx expression is highly cytotoxic to SH-SY5Y, immortalized HEK293 human kidney epithelial and immortalized ST14A striatal neuronal progenitor cells, when compared to NF-YAl or NF-YAs. Furthermore, NF-YAx induces KIF1Bβ-mediated necroptosis in these p53/p63/p73 compromised cell lines [35]. This suggests that transient NF-YAx expression during murine embryonic stages E12.5 through E14.5 could be involved in KIF1Bβ-dependent sympathetic neuroblast culling during this developmental stage [35,97].

In contrast to _DN_NF-YA, which is highly cytotoxic to SH-SY5Y cells, abrogates NF-Y CCAAT-box binding activity [73] and fails to select stable transfectants [35], NF-YAx does not eliminate all SH-SY5Y transfectants, permitting the propagation of stable transfectants in which NF-YAx readily substitutes other NF-YA isoforms in NF-Y complexes with CCAAT-box binding activity [35]. These stable transfectants do not express KIF1Bβ and exhibit a more stem cell-like gene expression pattern, characterized by enriched Sox2, CD117, p75NTR, Nestin and Nanog mRNA expression, compared to stable NF-YAl and NF-YAs SH-SY5Y transfectants. This suggests that NF-YAx selects a resistant more CSC-like subpopulation. In support of this, NF-YAx transfectants are considerably more resistant to doxorubicin-induced death than either stable NF-YAl or NF-YAs SH-SY5Y transfectants. This unveils a potential doxorubicin-induced, NF-YAx-mediated mechanism for cytotoxic selection of doxorubicin-resistance. Along this vein, SH-SY5Y cells engineered to express the NB-associated oncoprotein TrkAIII, which also exhibit a CSC-like phenotype, and exhibit enhanced tumorigenic and metastatic activity [98,99] but do not express NF-YAx, are also significantly more resistant to cytotoxicity induced by forced NF-YAx expression. This adds to the potential association between CSC-like phenotypes and resistance to NF-YAx cytotoxicity in non-*MYCN* amplified SH-SY5Y cells [35]. This suggests that NF-YAx could be a potential marker not only of response to doxorubicin but also the acquisition of doxorubicin resistance. This possibility is of potential prognostic and therapeutic significance, and may also help to explain the exclusive relatively high-level expression of NF-YAx mRNA in advanced stage 3 NB, and minor levels of NF-YAx mRNA expression in stage 2 NBs [35]. Although NF-YAx overexpression clearly enhances SH-SY5Y resistance to doxorubicin in vitro, this remains to be confirmed in appropriate in vivo models.

In contrast to NF-YAx transfectants, NF-YAl SH-SY5Y transfectants, which exhibit approximate NF-YAl to NF-YAs protein ratios of 98% to 2%, express lower levels of SOX2, p75, CD117, Nestin and Nanog mRNAs, supporting a more differentiated, less stem-cell-like phenotype. On the other hand, NF-YAs transfectants, which exhibit approximate NF-YAl to NF-YAs protein ratios of 2% to 98%, exhibit a more stem-cell-like gene expression pattern characterized by enhanced Sox-2 and Nanog mRNA expression but express lower levels of CD117, p75 and Nestin mRNAs than NF-YAx transfectants. These NF-YA isoform transfectants do not differ in proliferation rates in vitro. However, NF-YAl SH-SY5Y transfectants are significantly less tumorigenic in nude mice compared to both NF-YAs and NF-YAx SH-SY5Y transfectants, and are therefore potentially less aggressive [35]. We stress that these observations refer to the effects of NF-YAx in a single non-Myc amplified NB (SH-SY5Y) cell line, and in immortalized human kidney epithelial (HEK293) and striatal neural stem cell/progenitor (ST14A) cell lines. It remains to be determined whether the cytotoxic and selective action of NF-YAx extends to *MYCN* amplified NB cells.

**Figure 4:**
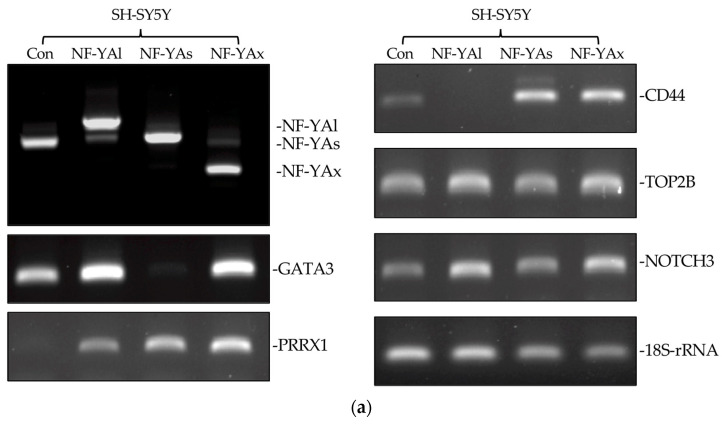

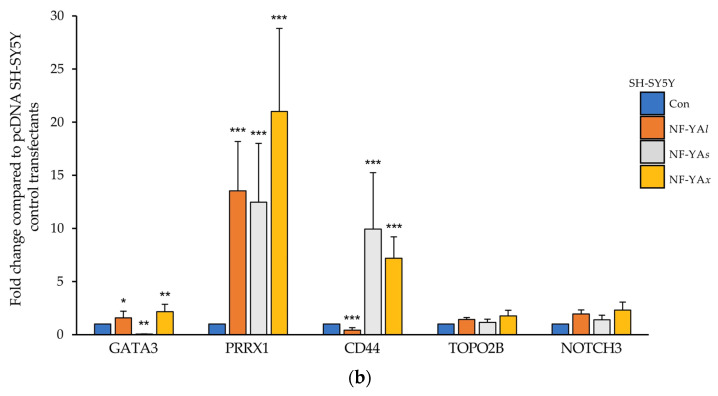
(**a**) Representative agarose gel of RT-PCRs plus (**b**) histogram demonstrating significantly enhanced expression of GATA3 and PRRX1 mRNAs and significant repression of CD44 mRNA in NF-YAl SH-SY5Y transfectants; significant repression of GATA3 mRNA and significantly enhanced PRRX1 and CD44 mRNAs in NF-YAs SH-SY5Y transfectants; and significantly enhanced expression of GATA3, PRRX1 and CD44 mRNAs in NF-YAx transfectants, compared to control SH-SY5Y transfectants, plus subtle non-significant changes in TOP2B and NOTCH3 mRNA levels in control, NF-YAl, NF-YAs and NF-YAx SH-SY5Y transfectants, in comparative densitometric analyses from 3 independent RT-PCR experiments performed in duplicate. (cDNA concentration for PCRs: 50ng for GATA3 and CD44; 0.5ng for PRXX1 and TOP2B; 5ng for NOTCH3) (* *p* = 0.048, ** *p* = 0.002, *** *p* < 0.0001) (see Supplementary Information: Figure S1 and File S1 for details) [35,103,104].

With regard to the potential influence of predominant NF-YAl, NF-YAs and NF-YAx expression on SH-SY5Y adrenergic/mesenchymal phenotype identity, we have now conducted preliminary semi-quantitative RT-PCR evaluations of mRNA expression for the constitutive adrenergic core regulator GATA3 [12,33,39,100,101], the mesenchymal core regulator PRRX1 [12,26,45,46], the adrenergic phenotype maintainer TOP2B [44], the mesenchymal phenotype and NCSC marker CD44 [12,23,26,31,47], and the promoter of adrenergic-to-mesenchymal conversion NOTCH3 [33,102] (methods and primer sets are described in Supplementary File S1) [35,103]. In control SH-SY5Y transfectants, which exhibit approximate NF-YAl/NF-YAs protein ratios of 40 to 60% [35], these experiments reveal a gene expression pattern characterized by relatively high constitutive mRNA expression of GATA3, TOP2B and NOTCH-3, and relatively low constitutive expression of CD44. In contrast, PRRX1 mRNA expression is undetectable. This would suggest a less NCSC-like proliferating adrenergic phenotype, which has been previously reported for this cell line [28,50]. NF-YAl SH-SY5Y transfectants express significantly higher levels of GATA3 (*p* = 0.048, *n* = 6) and PRRX1 (*p* < 0.0001, *n* = 6) mRNAs, and similar levels of TOP2B and NOTCH-3 mRNAs but significantly lower levels of CD44 mRNA (*p* < 0.0001, *n* = 6), compared to control transfectants. This suggests that predominant NF-Yl expression may promote a less tumorigenic proliferating intermediate hybrid adrenergic/mesenchymal-phenotype with interconverting potential and low NCSC identity. NF-YAs SH-SY5Y transfectants express significantly lower levels of GATA3 (*p* = 0.002, *n* = 6), significantly higher PRRX1 (*p* < 0.0001, *n* = 6) and CD44 (*p* < 0.0001, *n* = 6), and similar levels of TOP2B and NOTCH3 mRNAs, compared to control transfectants. This suggests that predominant NF-YAs expression may promote a more tumorigenic mesenchymal phenotype with NCSC identity. NF-YAx SH-SY5Y transfectants, which exhibit approximate NF-YAx/NF-YAl/s protein ratios of 99 to 1%, in contrast to NF-YAs transfectants, express significantly higher levels of GATA3 (*p* < 0.0001, *n* = 6), in addition to significantly elevated PRRX1 (*p* < 0.0001, *n* = 6) and CD44 (*p* < 0.0001, *n* = 6) expression, and similar levels of TOP2B and NOTCH3 mRNAs, compared to control transfectants (Figure 4). This expression pattern, combined with previous observations that NF-YAx transfectants exhibit increased Sox2, CD117, p75, Nestin and Nanog mRNA expression, compared to the other transfectants, and are doxorubicin-resistant [35], suggests that NF-YAx expression, in addition to promoting KIF1Bβ-induced necroptosis, also could select a doxorubicin-resistant tumorigenic subpopulation exhibiting a potentially hybrid mesenchymal/adrenergic phenotype with marked NSCS identity. We speculate that this subpopulation may have enhanced self-renewal and plasticity potential. This further supports the possibility that detection of predominant or exclusive NF-YAx expression in advanced stage and post-therapy relapsed NBs may have potential use as a marker of response to doxorubicin and also doxorubicin-resistance. We stress, however, that these proposed phenotype associations inferred by differences in mRNA expression of the master phenotype plasticity regulators GATA3 and PRRX1, and stemness-associated CD44 (this article), and Sox2, CD117, p75, Nestin and Nanog genes [35], are at present preliminary and must be confirmed at the protein level. NF-YAl SH-SY5Y transfectants, however, are significantly less tumorigenic in vivo than NF-YAs and NF-YAx SH-SY5Y transfectants [35], supporting a function in suppressing tumorigenesis. Furthermore, NF-YAx SH-SY5Y transfectants are significantly more resistant to doxorubicin than NF-YAl or NF-YAs SH-SY5Y transfectants [35], supporting a function in regulating sensitivity to doxorubicin through selection. It remains to be determined whether NF-YAx expression in SH-SY5Y cells is also induced by alternative DNA damaging chemotherapeutic agents, such as cisplatin or etoposide, and future studies will focus on more detailed transcriptome analysis and how different NF-YA isoforms influence the invasive and migratory activity of SH-SY5Y cells.

GATA-3, TOP2B, Sox2, Nanog and CD44 are all recognized to be NF-Y-regulated genes, whereas evidence is lacking for a direct role of NF-Y in PRRX1, NOTCH3, p75, CD117 and Nestin transcription [100,101,102,105,106,107,108,109]. These observations, therefore, support a functional difference in NF-Y complexes predominated by NF-YAl and NF-YAs with respect to GATA3 expression (high in NF-YAl transfectants and low repressed in NF-YAs transfectants) and CD44 (repressed in NF-YAl transfectants and high in NF-YAs transfectants). However, differences in GATA3 expression in NF-YAs (repression) and NF-YAx (high expression) are difficult to explain at present, as both variants exhibit exon 3 skipping and cannot, therefore, be explained by the loss of exon 3 sequence alone. Furthermore, similar GATA3 mRNA expression by fully spliced NF-YAl and exon 3- and 5-skipped NF-YAx transfectants indicates that loss of both exon 3 and 5 sequences does not repress but enhances GATA3 expression. This suggests that NF-YAs repression of constitutive GATA3 expression in SH-SY5Y cells depends upon the loss of exon 3 but presence of the exon 5 sequence. This is the subject of ongoing research. CD44 repression in NF-YAl transfectants, compared to relatively high expression in NF-YAs and NF-YAx transfectants, suggests that NF-Y complexes dominated by NF-YAs and NF-YAx isoforms with Q-rich truncations that result in smaller transactivation domains are potentially more efficient at inducing CD44 expression. The relatively high PRRX1 expression exhibited by NF-YA isoform transfectants, compared to control transfectants, implicates NF-YA overexpression, whereas the subtle non-significant variations in transfectant TOP2B and NOTCH3 expression suggest that NF-Y complexes pre-dominated by different NF-YA isoforms have little influence on their expression in SH-SY5Y cells (Figure 4 and Table 1).

Mechanisms through which NF-YAs and NF-YAx alter NF-Y function depend upon deletions in the NF-YA Q-rich transactivation domain. Such deletions would be expected to modify NF-Y transactivating potential and function as a pioneer transcription factor, by altering the role played by this domain in protein–protein interactions. Changes in these interactions would impact coactivator recruitment required for chromatin accessibility. The NF-YA Q-rich domain is a primary docking site for basal machinery, histone modifiers (e.g., p300 histone deacetylase) and SW1/SNF remodeling complexes, that could be modified by truncations. In addition, NF-Y also positions nucleosomes around CCAAT-boxes [58,59,60,61,62,63,64,65,66,67,68,69,70,71,72,73,74,75,76,77,78,79,80,81,82,83]. This suggests that Q-rich domain truncations could alter chromatin accessibility at key adrenergic and mesenchymal loci. NF-Y maintains transcriptionally active sites open by promoting chromatin accessibility and, therefore, is a fundamental regulator of Core regulatory circuitries. Truncations in the Q-rich domains of NF-YAs and NF-YAx may, therefore, modify chromatin accessibility by altering the recruitment of complexes that establish H3K27ac marks, modifying core adrenergic regulatory gene expression. With respect to mesenchymal loci, adrenergic-to-mesenchymal transitions are characterized by the substitution of one core regulatory network with another. Within this context, truncations in NF-YAs and NF-YAx Q-rich domains may modify NF-Y capacity to drive canonical gene expression, tipping the balance to repress or coordinate alternative mesenchymal programs, by silencing the expression of adrenergic genes or by altering access to genes of the mesenchymal lineage. With respect to our own analyses, NF-YAl and NF-YAs both bind Sp-1, whereas NF-YAx does not bind Sp1, due to additional truncation of the Q-rich Sp1/3 binding site from amino acids 55–139 in NF-YAs to amino acids 55–103 in NF-YAx [35]. Considering that NF-Y and Sp1 are both involved in NB cell neuronal differentiation [109], this may help to explain why NF-YAx transfectants exhibit an mRNA expression pattern, suggesting a potential hybrid more mesenchymal/less adrenergic phenotype with NCSC identity. Although speculative, NF-YAx may also have lost capacity to bind ZHX repressors, involved in differentiation from mesenchymal stem cell states [110], as the ZHX binding site in NF-YA overlaps that of Sp1 [111], and is also truncated in NF-YAx [35], but this remains to be confirmed. Furthermore, NF-YAx readily competes with NF-YAl in forming CCAAT box-binding NF-Y complexes with NF-YB and NF-YC in vitro, and CCAAT box-binding NF-Y complexes in stable NF-YAx SH-SY5Y transfectants are exclusively composed of NF-YAx, indicating that transcription regulation would be under almost exclusive NF-YAx control [35]. Although differences in the mRNA expression of core adrenergic, mesenchymal regulator genes and genes associated with stemness characterize NF-YAl, NF-YAs and NF-YAx SH-SY5Y transfectants, functional differences require further investigation at the transcriptome level.

The molecular mechanisms involved in regulating interconverting phenotype plasticity in NBs are highly complex. However, single-cell technologies with high-throughput lineage tracing have unified enhancer reprogramming, epigenetic plasticity, super-enhancer networks, and transcriptional state switching into a single paradigm, in which NBs grow through reversible, non-mutational epigenetic plasticity [20,25,112,113,114,115]. In this paradigm, NB cells shift between distinct transcriptional states by dismantling and rebuilding Core regulatory circuitries, rewiring super-enhancer networks to drive phenotype switching and therapeutic resistance. Subsequent phenotypic heterogeneity is driven primarily by transitions between two super-enhancer-defined cell states, the sympathoadrenal lineage committed adrenergic phenotype, driven by master transcription factors such as *GATA3* [46,101], and the undifferentiated, neural-crest-like mesenchymal state controlled by transcription factors, such as *PRRX1* [30,31]. Cell identity is locked in by dense super-enhancer loops that regulate specific master transcription factors. When NB cells switch phenotype, they undergo comprehensive enhancer reprogramming. Super-enhancer landscapes are completely reorganized, effectively turning off old lineage core regulatory circuits while activating new super-enhancers to alter the transcriptome, driven by highly reversible epigenetic modifications. This plasticity enables NB cells to adapt to stress within the tumor microenvironment, including therapies, facilitating therapeutic escape and subsequent relapse. Furthermore, as mentioned above, single-cell multi-omics profiling has also revealed an intermediate noradrenergic progenitor cell phenotype with extensive epigenetic priming that may act as a potential bridge for full bi-directional adrenergic-to-mesenchymal plasticity [12,25]. NF-YA splicing could fit into this scenario as a functional modifier of pioneer transcription factor NF-Y’s capacity to regulate chromatin accessibility to Core regulatory circuitries [59,60,70]. Within this context, NF-Y complexes predominated by NF-YAs, essential for maintaining embryonic stem cell Core regulatory circuitry, switch to complexes predominated by NF-YAl during embryonic stem cell differentiation that must suppress stem cell Core regulatory circuitries maintained NF-YAs, and activate Core regulatory circuits that drive differentiation. This is paralleled in neuronal progenitors and NB cells that switch from NF-YAs to NF-YAl expression during neuronal differentiation [73,78,79,80]. This implicates NF-YAs in less-differentiated neuronal progenitor and NB cell phenotypes and NF-YAl in more-differentiated neuronal and NB phenotypes. However, this contrasts to the effect of NF-YAs to NF-YAl switching in claudin-low breast cancer cells, which promotes EMT, indicating that NF-Y complexes predominated by NF-YAs or NF-YAl in NB and breast cancer cells may exhibit cell-type specific phenotype regulating effects [92].

In non-*MYCN* amplified adrenergic SH-SY5Y cells, which exhibits an approximate NF-YAl/NF-YAs protein expression ratios of 40 to 60% [35], stable transfection of NF-YAl, resulting in an NF-YAl/NF-YAs protein expression ratios of approximately 98 to 2%, results in a less tumorigenic phenotype and mRNA expression suggesting movement towards a hybrid adrenergic/mesenchymal phenotype with reduced NCSC identity (Figure 4 and Table 1). Stable transfection of NF-YAs, resulting in NF-YAl/NF-YAs protein expression ratios of approximately 2 to 98%, results in a tumorigenic phenotype and mRNA expression suggesting movement towards a potentially de-differentiated more mesenchymal, less-adrenergic phenotype with increased NCSC identity (Figure 4 and Table 1), whereas stable transfection of NF-YAx, resulting in an NF-YAx/NF-YAl/s protein expression ratio of approximately 99%/1%, results in a tumorigenic phenotype and mRNA expression suggesting movement towards a mesenchymal, less adrenergic phenotype with even more pronounced NCSC identity (Figure 4 and Table 1). Although speculative, this suggests that the similar levels of NF-YAl and NF-YAs mRNA expression detected in primary stage 1 and 2 NBs [35] may indicate less aggressive, more adrenergic phenotypes, NF-YAx expression in stage 2 NBs [35] may reflect a response to therapy, and high-exclusive NF-YAx mRNA expression in the stage 3 NB [35] could indicate a resistant mesenchymal, less adrenergic phenotype with pronounced NCSC identity, potentially induced by therapy. In contrast, detection of both predominant NF-YAl and predominant NF-YAs expression in stage 4 NBs, in the absence of NF-YAx, may suggest elevated adaptive plasticity capable of promoting either more-differentiated adrenergic or less-differentiated mesenchymal phenotypes.

Although the relatively small 30 NB cohort used to analyze NF-YA expression demonstrates that NF-YAx is expressed in a subpopulation of stage 2 and stage 3 NBs, this cohort is too small for extrapolation. Future analysis of NF-YA splicing must, therefore, be assessed in larger cohorts in order to more accurately establish how alternative NF-YA splicing, and in particular NF-YAx expression, associates with amplified and non-amplified *MYCN* status, disease stage, response to therapy and outcome.

Initial data, however, demonstrate NF-YA mRNA expression in only ≈50% of this 30 NB cohort [35], suggesting that NF-YA may not represent a universal transcriptional protagonist in NB pathogenesis and progression. Furthermore, detection of NF-YAx expression in 2 out of 10 stage 2 NBs and 1 of 6 stage 3 NBs indicates that NF-YAx is selectively and not generally expressed in NBs. Considering that doxorubicin induces NF-YAx expression in SHSY5Y cells, this may reflect differences in NB samples taken from pre-therapeutic, therapeutic and post-therapeutic settings, which should be taken into account in future studies.

With respect to NF-YA splicing regulation in cancers other than NB, NF-YAx expression has yet to be detected in other cancer types. However, shifts in the NF-YAl/NF-YAs ratio towards NF-YAs have been reported in breast and lung cancer progression [116], whereas a subclass of more aggressive low claudin expressing breast cancers exhibit high NF-YAl expression and a mesenchymal phenotype prone to metastasis [92]. In lung cancers, a shift to NF-YAs expression up-regulates metabolic enzyme and cell-cycle gene expression, whereas high NF-YAl expression up-regulates a pro-migration expression signature, and high NF-YAs expression characterizes all lung squamous cell cancer subtypes [117]. With respect to embryonic tumors other than NB, to our knowledge, there are currently no reports of NF-YA splicing regulation in either nephroblastoma (Wilms tumor) or medulloblastoma.

## 4. Conclusions

**Figure 5:**
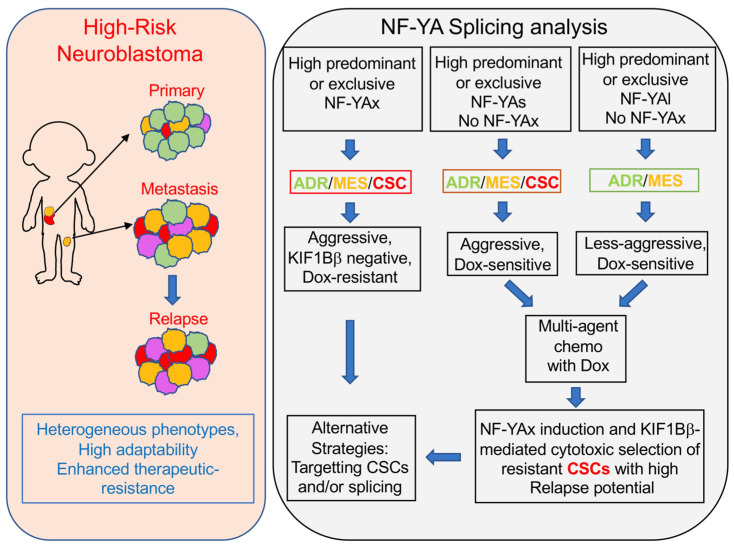
Proposed working model of the potential prognostic and therapeutic value of assessing alternative NF-YA splicing in NBs. Detection of dominant or exclusive NF-YAx expression in advanced stage or relapsed NBs may associate with a potentially more aggressive hybrid mesenchymal/adrenergic phenotype with NCSC-like identity, with enhanced resistance to doxorubicin (Dox) chemotherapy, and could, therefore, indicate worse prognosis. This may support the use of alternative strategies to target cancer stem cells (CSCs) or reverse NF-YAx splicing. Detection of predominant NF-YAl splicing in the absence of NF-YAx may indicate a less-tumorigenic adrenergic phenotype, whereas predominant NF-YAs expression in the absence of NF-YAx may indicate a potentially more-tumorigenic hybrid mesenchymal/adrenergic phenotype with NCSC identity (MES). Both of these conditions may be sensitive to doxorubicin but have potential to acquire resistance via doxorubicin-induced alternative NF-YAx splicing and the selection of NF-YAx expressing subpopulations that exhibit KIF1Bβ repression, eventually redirecting relapses for alternative strategies that target CSCs or NF-YAx splicing.

De-regulation of NF-Y function through alternative NF-YA splicing alters transcription factor networks, leading to changes in NB phenotypes that are likely to drive tumor adaptability within stressful TMEs [35,112]. Within this context, our observations unveil a possible double role for NF-YAx in cytotoxic responses to doxorubicin and in the selection of a doxorubicin-resistant subpopulation exhibiting a tumorigenic, less differentiated, more mesenchymal phenotype with NCSC identity. As a functional NF-Y modifier, NF-YAx replaces NF-YAl and NF-YAs in NF-Y complexes and has lost the capacity to bind Sp1/Sp3 transcription factors, which could potentially extend to ZHX transcription repressors [35]. This change in NF-Y function alters the equilibrium between proliferation, differentiation and sensitivity to doxorubicin. We speculate that NF-Y functional alterations caused by predominant NF-YAs or NF-YAx expression, by altering mRNA expression of key phenotype regulators, results in tumorigenic, less differentiated, more mesenchymal phenotypes with NCSC-identity. In contrast, NF-Y complexes predominated by NF-YAl that promote a less tumorigenic phenotype may do this by moving NB cells towards a more differentiated hybrid adrenergic/mesenchymal phenotype with low NCSC identity. In addition, NF-Y complexes dominated by NF-YAx induce KIF1Bβ expression and KIF1Bβ-mediated necroptosis in SH-SY5Y, HEK293 and ST14A cells, but select a resistant SH-SY5Y phenotype that does not express KIF1Bβ [35]. This also suggests a potential role for NF-YAx in the clinical heterogeneity of non-*MYCN* amplified NB. All three NF-YA isoforms, therefore, appear to be involved in regulating NB phenotypic plasticity and NSCS identity, with doxorubicin-induced NF-YAx expression unveiling an unexpected novel role for this isoform in doxorubicin cytotoxicity and acquisition of doxorubicin-resistance. Although these observations are preliminary, this possibility may be of relevance to NB post-therapeutic relapse and progression and, therefore, of eventual prognostic and therapeutic significance. It would seem reasonable, therefore, to analyze alternative NF-YA splicing in NBs, with particular focus on detecting predominant or exclusive NF-YAx expression, as a potential indicator of doxorubicin response and/or resistance, particularly in advanced stage or relapsed NBs. The identification of NBs exhibiting predominant or exclusive NF-YAx expression could potentially add to therapeutic stratification by singling out tumors that may benefit from alternative therapeutic approaches that exclude doxorubicin and target alternative NF-YAx splicing to induce expression of the NF-YAl isoform, which may promote a less-tumorigenic NB phenotype, with a lower NCSC-like component [1,118,119,120,121,122] (Figure 5).

The therapeutic use of splicing modulators to revert NF-YAx splicing back to NF-YAl is provocative and potentially clinically relevant but must take into account reports that NF-YAl may also promote less tumorigenic but more invasive phenotypes [92]. On the other hand, splicing modulators also inadvertently inhibit nonsense-mediated decay (NMD) to enhance tumor antigenicity that could be used to target NBs addicted to NMD [120]. However, the clinical use of splicing modulators has major challenges, including off-target toxicity and narrow therapeutic windows with potential to influence normal genes, resulting in unintended harmful protein isoforms and toxicities before achieving anti-tumor efficacy. In addition, tumors exhibit high interstitial fluid pressures and inadequate vasculatures reducing efficient delivery, and the high adaptability of NB cells could bypass inhibition by hijacking other splicing factors, or through rapid alterations in DNA methylation and epi-transcriptomics [121,122]. Problems with the biodistribution of splicing modulators, however, have prompted research into alternative chemistries and novel delivery systems, including the use of small molecules with excellent biodistribution properties or viral vectors to convey antisense sequences [123,124].

## Figures and Tables

**Table 1:** Proposed working model for possible phenotypes associated with dominant NF-YAl, NF-YAs and NF-YAx expression in stable SH-SY5Y transfectants, inferred by similarities and differences in GATA3, PRRX1, NOTCH3, TOP2B, CD44, p75, Sox2, CD133, CD117, Nestin and Nanog mRNA expression compared to control SH-SY5Y transfectants.

Stable Transfectants	Constitutive Expression	Significantly Increased Expression	Significantly Reduced Expression	Tumorigenicity In Vivo	Proposed Potential Phenotype
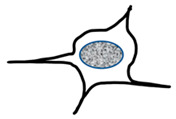 **pcDNA** **SH-SY5Y** NF-YAl/s ≈40/60%	**GATA3, PRRX1,** **NOTCH3,TOP2B** **CD44, p75,Sox2, CD133, CD117,** **Nestin, Nanog**			**Tumorigenic**	**ADRN**
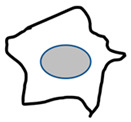 **NF-YAl** **SH-SY5Y** NF-YAl/s ≈98/2%	**NOTCH3,TOP2B** **p75, CD133,** **CD117, Nestin,**	**GATA3,** **PRRX1**	**CD44,** **Sox2,** **Nanog**	**Significantly** **less** **Tumorigenic**	**Hybrid** **ADRN/MES**
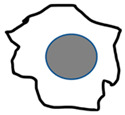 **NF-YAs** **SH-SY5Y** NF-YAl/s ≈2/98%	**NOTCH3, TOP2B, p75, CD133,** **CD117, Nestin**	**PRRX1,CD44,** **Sox2,** **Nanog**	**GATA3**	**Tumorigenic**	**Hybrid** **MES/ADRN** **NCSC**
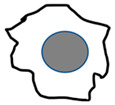 **NF-YAx** **SH-SY5Y** NF-YAx/L/s ≈99/1%	**NOTCH3,** **TOP2B,** **CD133**	**GATA3, PRRX1, CD44, p75,** **Sox2,** **Nestin, Nanog, CD117**		**Tumorigenic**	**Hybrid** **MES/ARDN** **NCSC**

## Data Availability

The data presented in this study are available from the corresponding author upon reasonable request.

## Supplementary Materials

The following supporting information can be downloaded at: https://www.mdpi.com/article/10.3390/cancers18111839/s1, Figure S1: Uncropped versions of agarose gel RT-PCRs presented in Figure 4; File S1: Cell culture and RT-PCR.

## Abbreviations

The following abbreviations are used in this manuscript:

NB	neuroblastoma
NF-Y	nuclear factor Y
TME	tumor microenvironment
EMT	epithelia mesenchymal transition
ADR	adrenergic
NOR	noradrenergic
NCSC	neural crest stem cells
CSC	cancer stem cell
PHOX2B	paired-like huomeobox-2B
ASCL1	Achaete-Scute family bHLH transcription factor-1
HAND1/2	heart and neural crest derivatives 1/2
TBX	T-box transcription factor
ISL1	insulin gene enhancer binding protein 1
GATA3	GATA binding protein 3
MYCN	v-myc avian myelocytomatosis viral related oncogene
TOP2B	DNA topoisomerase II-beta
PRRX1	paired related homeobox 1
AP-1	activator protein 1
IRF1-3	interferon related factor-1
RUNX1/2	runt-related transcription factor
MEOX1/2	mesenchyme homeobox i 2
SIX1/4	sine oculis homeobox
SOX	SRY-related HMG-box
SMAD	small worm phenotype-mothers against decapentaplegic
WWTR1	WW domain-containing transcription regulator-1
SNAI1	snai1 family transcriptional repressor 1
NOTCH3	Neurogenic locus notch homolog protein 3
PCNA	proliferating cell nuclear antigen
MCM4	microsomal maintenance complex component
CCNA1	cysteine-rich angiogenic inducer 61
CCNB1/2	cyclin B1
PLK	polo kinase
AURKB	aurora Kinase
CHOP	C/EBP homologous protein
XBP1	X-box binding protein-1
TP53	tumor protein 53
TP63	tumor protein 63
HIF1	hypoxia inducible factor-1
BIMI1	B cell-specific Moloney murine leukemia virus integration site-1
SREBP	sterol regulatory element-binding protein
HMGCS1	3-hydroxy-3-methylglutaryl-CoA synthase-1
HMGCR	3-hydroxy-3-methylglutaryl-coenzyme A reductase
SQLE	squalene epoxidase
DHCR7	7-dehydrocholesterol reductase
ACAC	acetyl-CoA carboxylase 1
FASN	fatty Acid Synthase
SCD	stearoyl-CoA desaturase
PFKFB21-3	6-phosphofructose-2-kinase
PGK1/2	phosphoglycerate kinase
GADPH	gylceraldehyde-3-phosphate dehydrogenase
PKM	pyruvate kinase Muscle
LDHA	lactate dehydrogenase
HK2	hexokinase-2
ALDOB	aldolase, fructose-bisphosphate B
PHGDH	phosphoglycerate dehydrogenase
PSAT1	phosphoserine aminotransferase1
PSPH	phosphoserine phosphatase
MTHFD2	methylenetetrahydrofolate dehydrogenase 2
DHFR	dihydrofolate reductase
GLS	glutaminase
AMD1	adenosylmethionine decarboxylase
OCD1	ornithine decarboxylase
MTAP	methylthioadenosine phosphorylase
AK2	adenylate Kinase 2
RRM1	ribonucleotide reductase catalytic subunit M1
RBM47	RNA binding motif protein 47
PTB1	polypyrimidine tract-binding protein 1
HuR	Hu antigen R
RBFOX2	RNA binding Fox-1 homolog
SOX2	sex-determining region Y (SRY)-box
CD117	cellular-KIT
CD44	cluster of differentiation 44
KIF1B	kinase family member 1B
