# Supplementary material for: Alternative Splicing of the NF-Y Subunit, NF-YA, in Neuroblastoma Phenotype Heterogeneity
Source: Cancers (Basel). 2026 Jun 4;18(11):1839. doi: 10.3390/cancers18111839 (PMC13257248; doi:10.3390/cancers18111839)
Supplement: Supplementary file 1 [file cancers-18-01839-s001.zip › Supplementary Figure S1 legend.pdf]

Supplementary Figure S1

Figure S1

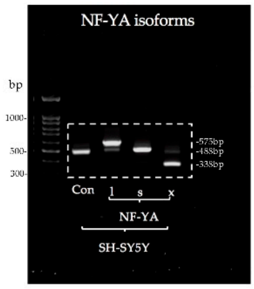

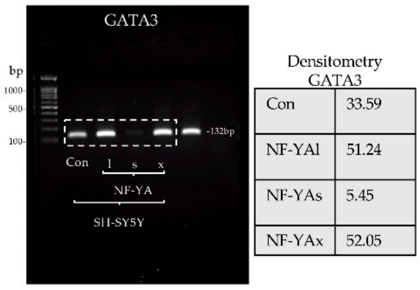

Densitometry GATA3

Con	33.59
NF-YAl	51.24
NF-YAs	5.45
NF-YAx	52.05

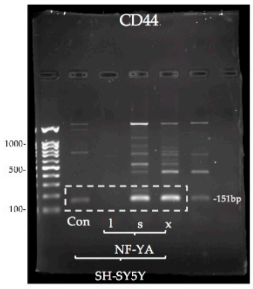

Densitometry CD44

Con	2.95
NF-YAl	0
NF-YAs	35.39
NF-YAx	37.16

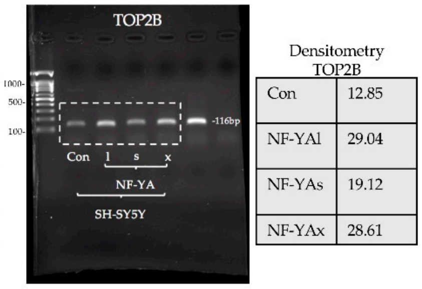

Densitometry TOP2B

Con	12.85
NF-YAl	29.04
NF-YAs	19.12
NF-YAx	28.61

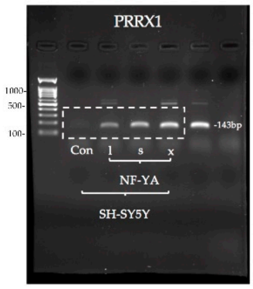

Densitometry PRRX1

Con	3.01
NF-YAl	17.07
NF-YAs	27.43
NF-YAx	21.09

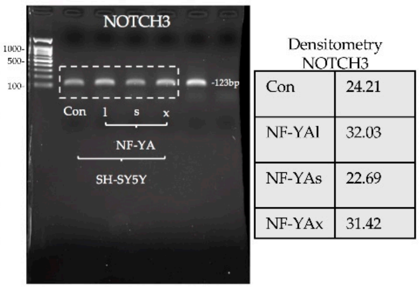

Densitometry NOTCH3

Con	24.21
NF-YAl	32.03
NF-YAs	22.69
NF-YAx	31.42

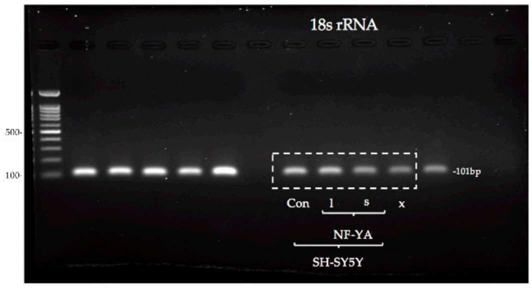

Densitometry 18s rRNA

Con	39.97
NF-YAl	39.69
NF-YAs	27.64
NF-YAx	21.09

Uncropped versions of agarose gel RT-PCRs presented in Figure 4, demonstrating NF-YAl (575bp amplicon), NF-YAs (488bp amplicon) NF-YAx (338bp amplicon), GATA3 (132bp amplicon), CD44 (151bp amplicon), TOP2B (116bp amplicon), PRXX1 (143bp amplicon), NOTCH3 (123bp amplicon) and 18s rRNA (101bp amplicon) RT-PCR products in RNAs purified from stable pcDNA SH-SY5Y (Con), NF-YAl SH-SY5Y, NF-YAs SH-SY5Y and NF-YAx SH-SY5Y transfectants (relevant RT-PCR products are highlighted in dashed boxes). Relative densitometric values of the RT-PCRs products for each gel are presented in the adjacent tables. The RNA quantities used for each RT-PCR were as follows: 50ng for GATA3 and CD44, 0.5ng for PRRX1 and TOP2B, 5ng for NOTCH3 and 0.05ng for 18s rRNA. Pixel densitometric values for each RT-PCR product, minus values from identical adjacent areas, were obtained for each individual band, using FIJI image J software [123].
