# Supplementary material for: Alternative Splicing of the NF-Y Subunit, NF-YA, in Neuroblastoma Phenotype Heterogeneity
Source: Cancers (Basel). 2026 Jun 4;18(11):1839. doi: 10.3390/cancers18111839 (PMC13257248; doi:10.3390/cancers18111839)
Supplement: Supplementary file 1 [file cancers-18-01839-s001.zip › Supplementary File S1.pdf]

Cell culture and RT-PCR

The parental SH-SY5Y NB cell line, obtained from Dr. U.P. Thorgeirsson (NCI, NIH, Bethesda, MD, USA), was used to obtain stable transfected pcDNA SH-SY5Y, NF-YAl SH-SY5Y, NF-YAs SH-SY5Y and NF-YAx SH-SY5Y cell lines [35]. Stable transfectants were cultured in RPMI 1649, supplemented with 10% Fetal Bovine Serum, 1% glutamine, 1% penicillin/streptomycin (Euroclone, Milan, Italy), and intermittently cultured in complete medium containing zeocin (200 ug/mL) (Thermo Fisher Scientific, Waltham, MA, USA).

For RT-PCRs, total cellular RNAs (1 µg) were purified from ≈80% confluent cell cultures using a Quick-RNA™ Miniprep Kit, as described by the manufacturer (Zymo Research, Freiberg im Breisgau, GE), and were reverse-transcribed using SuperScript™ IV VILO™ Master Mix, as described by the manufacturer (Thermo Fischer Scientific, Waltham, MA, USA). Linear sub-plateau phase PCR amplification was established for each gene by comparing cDNA serial dilutions ranging from non-diluted to 1 to 100,000, in 35 cycle reactions [124]. The final cDNA dilutions and volumes used in PCRs were as follows: 1µl of 1 to 100,000 dilution (0,0005 ng cDNA) for 18S rRNA; 1µl non-diluted (50 ng cDNA) for GATA3, CD44 and NF-YA; 1µl of a 1 to 10 dilution (5 ng cDNA) for NOTCH3; and 1µl of a 1 to 100 dilution (0.5 ng cDNA) for PRRX1 and TOP2B.

The primers (and PCR conditions) used are as follows: 18S rRNA: 5′-AAACGGCTACCACATCCAAG-3′ and 5′-CCTCGAAAGAGTCCTGTATTG-3′ (denaturation 30s at 94°C, annealing 30s at 58°C and extension 30s at 72°C); GATA3: 5′-ACCACAACCACACTCTGGAGGA-3′ and 5′-TCGGTTTCTGGTCTGGATGCCT-3′ (denaturation 30s at 94°C, annealing 30s at 60°C and extension 30s at 72°C); CD44: 5′-CCAGAAGGAACAGTGGTTTGGC-3′ and 5′-ACTGTCCTCTGGGCTTGGTGTT-3′ (denaturation 30s at 94°C, annealing 30s at 60°C and extension 30s at 72°C); NOTCH3: 5′-TACTGGTAGCCACTGTGAGCAG-3′ and 5′-CAGTTATCACCATTGTAGCCAGG-3′ (denaturation 30s at 94°C, annealing 30s at 60°C and extension 30s at 72°C); PRRX1: 5′-TGCAGGCTTTGGAGCGTGTCTT-3′ and 5′-CTCATTCCTGCGGAACTTGGCT-3′ (denaturation 30 s at 94°C, annealing 30s at 60°C and extension 30s at 72°C); TOP2B: 5′-GGTCAGTTTGGAACTCGGCTTC-3′ and 5′-AGGAGGTTGTCATCCACAGCAG-3′ (denaturation 30s at 94°C, annealing 30s at 60°C and extension 30s at 72°C); NF-YA: 5′-AATAGTTCGACAGAGCAGATTG-3′ and 5′-TCCTGCCAAACTGGCTGCTGGGAT-3′ (denaturation 30s at 94°C, annealing 30s at 56°C and extension 30s at 72°C). All PCRs were 35 cycles.

All RT-PCRs were performed in duplicate and repeated three times. For densitometry, jpeg images of agarose gels were analyzed by ImageJ pixel densitometric analysis (http:imagej.nih.gov) and densitometric values for RT-PCR products in NF-YA isoform transfectants, adjusted with respect to 18S rRNA RT-PCR (n=6 for each gene), were compared to densitometric values of corresponding RT-PCRs in control transfectants, adjusted with respect 18S rRNA RT-PCR, and expressed as mean ± s.d. fold difference with respect to pcDNA SH-SY5Y controls (n=6, arbitrary value of 1). The results were then statistically compared by Student's t test, using the online t-test calculator at https://www.graphpad.com/quickcal/ttest1.cfm. Significant differences were associated with probability p values ≤0.05.
