# Supplementary figures and images for: Alternative Splicing of the NF-Y Subunit, NF-YA, in Neuroblastoma Phenotype Heterogeneity
Source: Cancers (Basel). 2026 Jun 4;18(11):1839. doi: 10.3390/cancers18111839 (PMC13257248; doi:10.3390/cancers18111839)

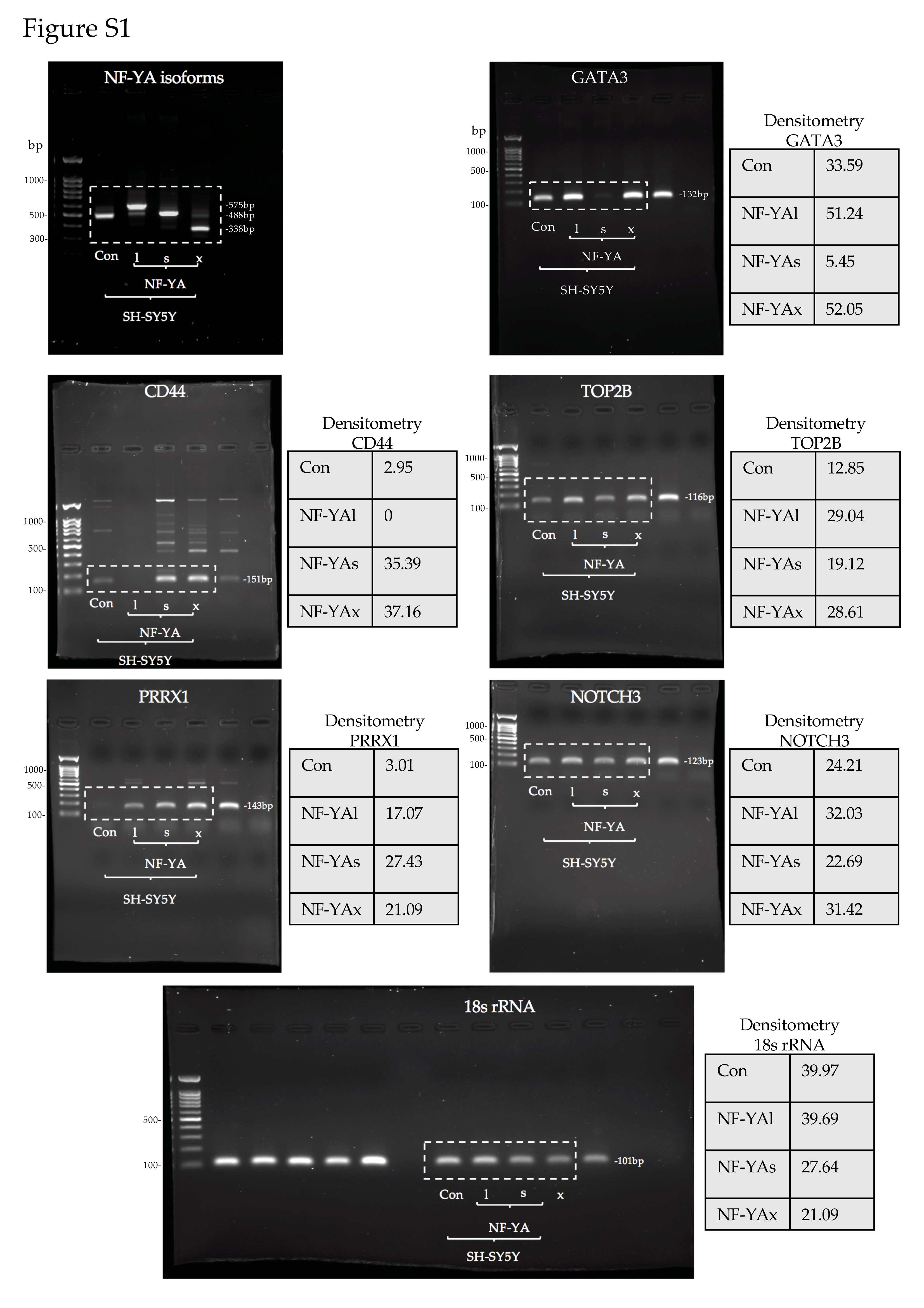

Supplement: Supplementary file 1 [file cancers-18-01839-s001.zip › Supplementary Figure S1.jpg]
